# Differential hydraulic and carbon economic strategies among four oak species

**DOI:** 10.3389/fpls.2026.1983948

**Published:** 2026-10-15

**Authors:** Qiang Li, Xinke Sun, Jiaqi Jiang, Zixuan Feng, Wenlong Sun, Yao Huang, Ning Du

**Affiliations:** 1 Key Laboratory of Tropical Translational Medicine of Ministry of Education, School of Public Health, Hainan Academy of Medical Sciences, Hainan Medical University, Haikou, China; 2 LTD of China Power Engineering Consulting Group, North China Power Engineering Co., Beijing, China; 3 Qingdao Key Laboratory of Ecological Protection and Restoration, School of Life Sciences, Shandong University, Qingdao, China; 4 Ministry of Education Key Laboratory for Genetics and Germplasm Innovation of Tropical Special Forest Trees and Ornamental Plants, Hainan University, Haikou, China

**Keywords:** carbon-water use strategies, Huber values, plant hydraulics, *Quercus*, stomatal size-density trade-off

## Abstract

**Background:**

The fluctuations of precipitation will become more erratic and dramatic, threatening the growth and survival of forest species. Plants usually integrate water and carbon management strategies to deal with environmental variations. The hydraulic strategies of woody plants would modify their carbon capture and allocation patterns, thus characterizing divergent strategies in water and carbon use economy could elucidate the differential responses to climate change among closely related species. To decipher possible correlations between functional traits relating to plant hydraulics and carbon economics, we selected four oak species (one native species, *Quercus dentata*, and three exotic species, *Q*. *rubra*, *Q*. *robur*, and *Q*. *palustris*) growing in an Oak Nursery to explore trait associations in hydraulic and carbon economics, and to contrast strategy variations among four species.

**Results:**

Our results showed a trade-off between leaf gas exchange rate and water use efficiency, where fast gas exchange related to fluctuating water potential. In carbon allocation patterns, higher Huber values could supply sufficient water in leaves, thereby maintaining water potential stable.

**Conclusions:**

Compared to the exotic oaks, *Q*. *dentata* adopted a relatively conservative strategy, embodying high water use efficiency, stable water potential, and leaves with smaller but denser stomata. However, *Q*. *rubra* was conservative and rapid-responsive, *Q*. *robur* was potentially drought-tolerant, and *Q*. *palustris* employed a strategy of less drought-resistant but might be more competitive in the face of abundant resources. Integrating these findings into global conservation efforts will lead to the development of adaptive management practices that foster tree species with better adaptation to environmental variations.

## 1 Introduction

Drought events are projected to become more frequent, intense and erratic, and the dramatic fluctuations of precipitation threaten the survival of forest species, causing large-scale mortality and changes in distribution patterns of trees (Aguirre-Gutiérrez et al., 2025; Bauman et al., 2022; Brodribb et al., 2020). Current and future changes in episodic precipitations have reportedly limited the carbon assimilation and growth of trees, suppressing the carbon sink capacity of forests (Yan et al., 2024). Characterizing the water- and carbon-use strategy of woody species is essential for predicting the future dynamics of forests and promoting resilient forest restoration as a nature-based solution to mitigate climate change (Guillemot et al., 2022).

Functional trait-based approaches have proven that functional constraints and trade-offs can underlie plant ecological strategies across species (Mueller et al., 2024). In addition, functional traits can help predict the effects of drought on forest dynamics (Aguirre-Gutiérrez et al., 2025, Guillemot et al., 2022). Specifically, plants must manage to survive extreme drought events by balancing water conservation and carbon uptake, entailing a trade-off between carbon assimilation vs stress tolerance (Cordero et al., 2021; Torres-Ruiz et al., 2024). The water and carbon management strategies generally include stomatal regulation and the associated plant water potential fluctuations, tissue/organ and whole-plant hydraulics and the associated water acquisition and reservation, and carbon utilization and allocation patterns (Johnson et al., 2018b). The interaction between the water relations and the carbon economy has been increasingly recognized as critical under extreme events, calling for the needs to be considered in forest function evaluation and mortality prediction (Martinez-Vilalta et al., 2019).

Drought-induced mortality is primarily ascribed to xylem hydraulic failure or to a combination of xylem hydraulic failure and carbon limitation (Guillemot et al., 2022; Li et al., 2020). Current knowledge emphasizes that stomata are the primary regulators balancing photosynthetic carbon gain and water loss (transpiration) to the atmosphere, and the ability to alter cell turgor adjusting leaf hydraulics could also ensure that leaves maintain photosynthesis under progressively drier conditions (Huang et al., 2018; Johnson et al., 2018a). Previous studies have proved the important role of stomatal anatomy (i.e. guard cell shape and size, stomatal density) and leaf hydraulics in determining the rapid responses of stomata (Meinzer et al., 2017). Besides, the key role of carbon allocation patterns in mediating plant hydraulics arise because of its relative plasticity. Specific leaf area (SLA, leaf area per unit of dry leaf mass) is tightly related to carbon allocation to leaf, which reflects resource acquisition strategy and further affects productivity (Lloret et al., 2018). The ratio of sapwood area to subtended leaf area (the Huber value, Hv) reflects not only the balance between water supply and loss, but also carbon allocation to stems vs leaves. Previous studies offered evidence that plants with low Hv tend to have high SLA, high carbon assimilation potential, and high hydraulic conductivity, pointing to fast resource economics and better competition performance (Xu et al., 2021).

Oaks (*Quercus* spp.) are a keystone genus in northern temperate zones, worldwide facing substantial challenges in natural recruitment and affecting biodiversity and ecosystem services (Franzén et al., 2024). Oaks differ greatly in distribution relative to the availability of water, but also behave well-adapted to drought (Cavender-Bares and Holbrook, 2001). Given their ecological dominance and remarkable abundance, oaks provide a critical means to study from individual leaves to whole ecosystems, and from individual populations to a globally distributed lineage (Cavender-Bares, 2016, Cavender-Bares, 2019). Many *Quercus* species have commonly demonstrated capacity for adaptive responses to climate change, suggesting they may be able to adjust responsive strategies as environments change (Rauschendorfer et al., 2022). Previous data showed that some *Quercus* species performed high flexibility in key functional traits in adapting to new environments (Cavender-Bares, 2019), yet there were also notable similarity in carbon allocation strategy and drought resilience for a given species in its natural habitat and induced environment (Wang et al., 2023). To better elucidate the association between hydraulic characteristics and carbon economics, it is essential to compare plant functional traits across closely-related species, therefore, to establish an intrinsic coordination of responses (Yao et al., 2021). However, a key gap remains: few studies have compared hydraulic and carbon-economic traits among closely related oak species growing in a common environment, especially between native and introduced oaks used in temperate afforestation. This limits our ability to identify the trait coordination and strategy trade-offs that underlie drought adaptation and species selection.

In order to establish associations between leaf hydraulic traits and gas exchange, we selected four oak species (*Q. dentata*, *Q. rubra*, *Q. robur* and *Q. palustris*) deliberately selected four oak species with contrasting life histories because we wanted to contrast trait variations in four species growing in the same environment. Notably, *Q. dentata* is native to China and dominant in warm-temperate forests, while *Q. rubra*, *Q. robur* and *Q. palustris* are exotic and have been widely introduced and cultivated for urban afforestation in China for decades. Notably, *Q. rubra* is native to North America, displays an anisohydric behavior and has been proven successfully adapted to Swedish climate (Thomsen et al., 2013; Franzén et al., 2024). *Q*. *robur* is a major oak species in Central Europe, considered to be less drought-sensitive and highly hygrophilous (Morecroft and Roberts, 1999). *Q. palustris* is widely distributed in North America, typically wet-adapted and hygrophilous (Kusi and Karsai, 2020). We were interested in understanding trait coordination and strategy variation among closely-related species. We hypothesized that (i) fast leaf gas exchange is coordinated with efficient plant hydraulics, and (ii) exotic oaks are more efficient in hydraulics and carbon economics, but less drought-resistant than the native oak species *Q. dentata*.

## 2 Materials and methods

### 2.1 Site and plant material

This study was conducted at an Oak Nursery in Qingdao, Shandong, China (36.587° N, 120.210° E, approximately 54.5 m a.s.l.), with a temperate monsoon climate, and > 70% of the total annual precipitation occurs in the areas of collection as rainfall during the growing season from May to September. Acorns were purchased from Dadi Agriculture CO., LTD, Shuyang, Jiangsu, China (34.137° N, 118.798° E; approximately 37 m a.s.l.). According to the supplier, acorns were collected from single natural population of each species in Dongtai, Jiangsu, China (32.349° N, 120.157° E, approximately 5.1 m a.s.l.). Acorns were sown manually in March 2015 at a depth of 4~5 cm in 50-cell plug trays filled with mixed sandy loam. Seedlings were transplanted to the common garden in July 2015 at a spacing of approximately 2 m. All saplings were well-watered and fertilized to ensure favorable conditions during the nursery period. Five-year-old saplings were used and the average size for the studied individuals was 3.53 ± 0.26 m in height and 5.95 ± 0.20 cm in diameter at breast height (DBH) for *Q. dentata*, 3.78 ± 0.14 m in height and 6.46 ± 0.17 cm in DBH for *Q. rubra*, 4.19 ± 0.34 m in height and 5.11 ± 0.24 cm in DBH for *Q. robur*, and 4.44 ± 0.09 m in height and 5.25 ± 0.08 cm in DBH for *Q. palustris* (see **Supplementary Table S1**). Fully-expanded and healthy sun leaves were selected from eight individuals of each species, and leaves were sampled from the top of the canopy. Sampling was conducted in August 2020, and all the measurements were finished in two weeks. For each trait, we sampled eight replicates for each species.

### 2.2 Leaf gas exchange

Leaf gas-exchange measurements were performed on 16th August 2020 (a sunny day) between 09:00 and 11:00 h. Eight individuals per species were measured. Stomatal conductance (*g*_s_, mmol m^-2^ s^-1^), net photosynthetic rate (*A*, μmol CO_2_ m^-2^ s^-1^), and transpiration rate (*E*, mmol m^-2^ s^-1^) were determined *in situ* from 09:00 to 11:00 h on a sunny day with a portable gas exchange system (Li-6800; Li-Cor, Lincoln, NE, USA). During measurements, photosynthetic photon flux density inside the leaf chamber was 1200 μmol m^-2^ s^-1^, leaf chamber CO_2_ concentration was 400 μmol mol^-1^, and average chamber air temperature was maintained at 28 °C. Based on the measurements, instantaneous water use efficiency (iWUE) was calculated as the ratio of *A* to *E*.

### 2.3 Plant hydraulics

Plant hydraulic measurements were conducted in the lab during 1^st^-10^th^ August 2020. Predawn and midday water potentials were measured on during 1^st^-3^rd^ August between 03:00-05:00 h and 12:00-14:00 h, respectively. Pressure-volume curves were measured on 5^th^-10^th^ August. The measurements of hydraulic traits for all four species × eight replicates took 10 days in total. Water potentials were measured using a pressure chamber (1505D-EXP; PMS Instruments, Albany, OR, USA). Leaf predawn (Ψ_s, pd_, MPa) and midday water potential (Ψ_s, md_, MPa) were measured at 03:00-05:00 h and 12:00-14:00 h, respectively. As the predawn leaf water potential is usually assumed to be near-equilibrium with overnight soil water potential for plant individuals growing in non-extreme conditions, we calculated the difference of leaf water potential between predawn and midday (Ψ_pd-md_, MPa) to estimate daily water potential fluctuation for an individual.

For pressure-volume (PV) curve measurement, small leafy shoots were excised and recut immediately under water in the early morning (05:00-06:00 h), before sealing in three layers of plastic bags. The sealed shoots were then taken back to the lab and rehydrated in darkness for 2-3h before PV curve measurements. After rehydration, the saturated weights and initial water potentials were recorded. And then the shoots were allowed to dry slowly on the laboratory bench while weights and water potentials were recorded periodically. Finally, the shoots were oven-dried at 70 °C for 72 h to constant weight for dry mass measurements. We then used the Sack & Pasquet-Kok PV_analysis spreadsheet tool to fit pressure-volume curves, where the turgor loss point (TLP, MPa) was extracted. The PV_analysis spreadsheet tool fits lines by standard major axis, and the data analyses including testing and correcting for “plateau effects” to make the parameter calculation more robust.

Upper canopy branches were excised for sapwood and leaf measurements. Cross-section sapwood area (SWA, cm^2^) was determined by measuring total xylem area of stained (safranin red) 0.5-1.0 cm thin sections using ImageJ 1.51j (National Institutes of Health, Bethesda, MD, USA). Distal leaves were hydrated to determine fresh weight, and then scanned for determination of leaf area (LA, m^2^) using ImageJ software. After leaf area determination, leaf samples were oven-dried at 70 °C for 48 h then weighed for dry mass. Leaf relative water content (RWC_leaf_) was calculated as the ratio of the difference between fresh weight and dry mass to dry mass. As an indication of hydraulic architecture, the Huber value (Hv, m^2^ m^-2^) was calculated as LA: SWA. The specific leaf area (SLA, cm^2^ g^-1^) was calculated as the ratio of LA to dry mass.

### 2.4 Stomatal anatomy

The epidermal impression technique was applied for anatomical analyses of stomata. Clear nail polish was used to cover the abaxial surface of one leaf per individual, and then peeled off to mount onto microscopy slides. Leaf imprints were collected on 16^th^ August from eight plants per species. One fully expanded sun leaf per individual was sampled, and 3 imprints were taken per leaf. For each species, eight individuals were sampled. Thus, a total of 96 imprints were collected. All slides were then observed with a light microscope (CX31RTSF, Olympus, Tokyo, Japan), and photographed at ×100 magnification to calculate stomatal density (SD, mm^-2^), and at ×400 magnification for the measurements of guard cell length (GL, µm) and width (GW, µm). Picture analyses were done in AJ-VERT software (X64, AOR Industrial CO., LTD, Shenzhen, China). Additionally, the stomatal area (SA, µm^2^) was calculated as:


$$SA=3.14\times \frac{1}{4}\times \left(GL\times GW\right)$$

### 2.5 Data analysis

One-way analysis of variance (ANOVA) was performed to test the difference among four species for all measured traits. The Kaiser Meyer Olkin test (KMO = 0.66) and Bartlett’s Test of Sphericity (*p* < 0.001) were conducted before Principal components analysis (PCA) was performed, and the PC axis variable loadings were assessed the correlation between the original variable and a certain principal component variable to evaluate key traits for four species. Before analysis, data were checked for normality and homogeneity. All data analyses were conducted using the Origin 2022 software package (OriginLab, MA, USA).

## 3 Results

### 3.1 Plant functional traits among four oak species

We applied ANOVA on all the measured functional traits to detect differences between four species. For leaf gas exchange traits, leaf transpiration rate (*E*) and net photosynthetic rate (*A*) were significantly higher in *Q. robur* and *Q. palustris* than those in *Q. dentata* and *Q. rubra* (**Supplementary Table S2**). Notably, instantaneous water use efficiency (iWUE) of *Q. palustris* was lower than those of the other three oaks species (**Supplementary Table S2**; **Figure 1A**). The significant difference in stomatal conductance (*g*_s_) only showed between *Q. rubra* and *Q. palustris* (**Supplementary Table S2**; **Figure 1B**). Net photosynthetic rate (A) was significantly higher in Q. robur and Q. palustris than in Q. dentata and Q. rubra (**Supplementary Table S2**).

**Figure 1:**
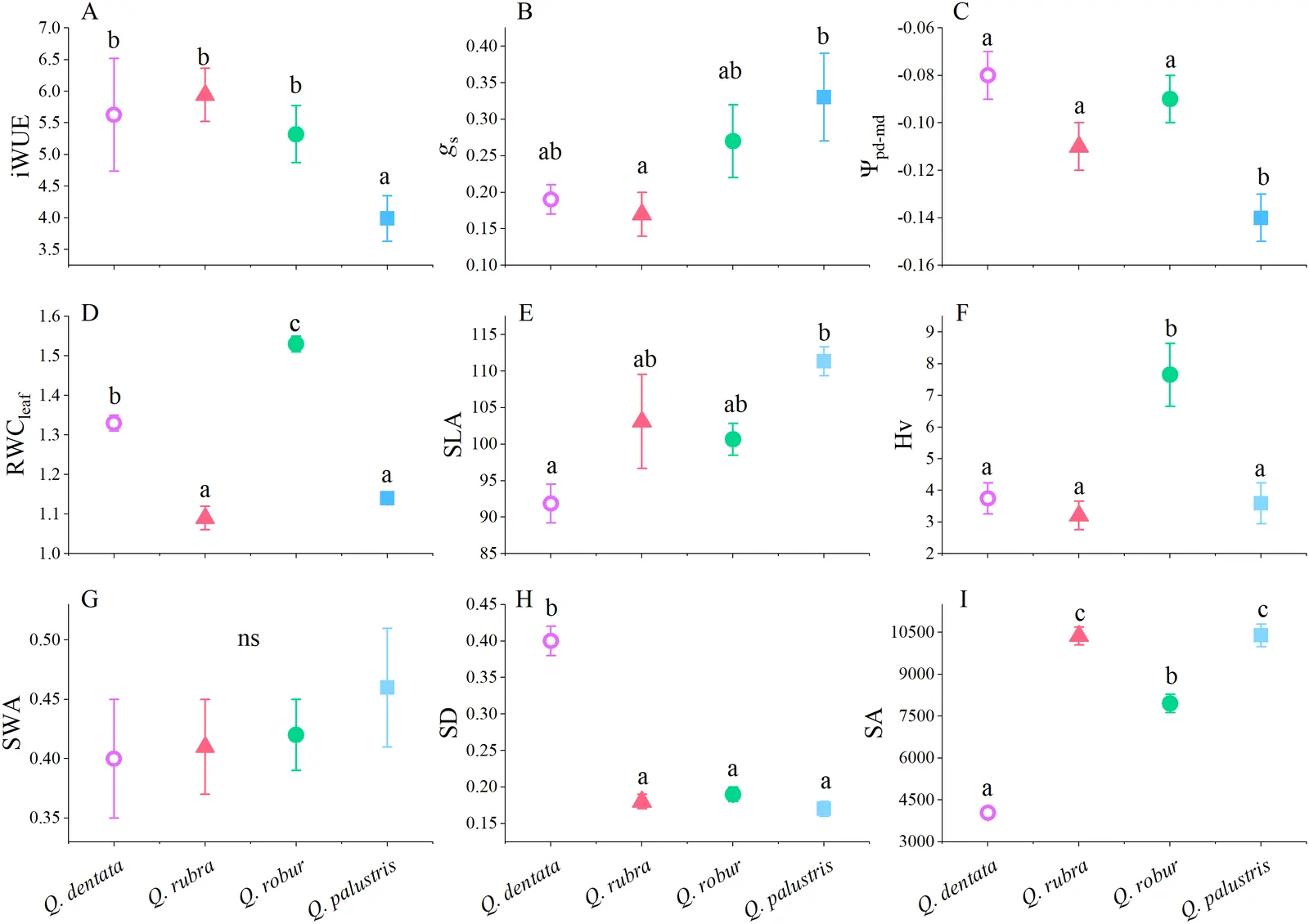
Scatter chart for key plant functional traits (**A**, iWUE; **B**, gs;**C**,Ψpd-md; **D**, RWCleaf; **E**, SLA; **F**, Hv; **G**, SWA; **H**, SD; **I**, SA) among four oak species. Data are shown in mean±SE and letters are shown when significant differences between treatments were detected (P<0.05).Trait abbreviations are given in **Figure 2**.

For hydraulic traits, no significant differences were found in osmotic potential at the turgor loss point (Ψ_TLP_) among the four oak species, while Ψ_pd-md_ was lower (more negative) in *Q. palustris* significantly and Huber value (*H*_v_) was higher in *Q. robur* significantly (**Supplementary Table S2**; **Figures 1C, F**). Leaf relative water content (RWC_leaf_) in *Q. rubra* and *Q. palustris* was not different, but lower than that in *Q. dentata*, while RWC_leaf_ in *Q. robur* was significantly higher than other three species (**Supplementary Table S2**; **Figure 1D**).

For structural traits, specific leaf area (SLA) was significantly lower in *Q. dentata* but significantly higher in *Q. palustris* than in *Q. rubra* and *Q. robur* (**Supplementary Table S2**; **Figure 1E**). Stomatal density (SD) was significantly higher in *Q. dentata* but among the other three species (**Supplementary Table S2**; **Figure 1H**). Stomatal area (SA) was significantly lower in *Q. dentata* but significantly higher in *Q. rubra* and *Q. palustris* (**Supplementary Table S2**; **Figure 1I**). Both guard cell length (GL) and guard cell width (GW) were significantly lower in *Q. dentata* than other three species (**Supplementary Table S2**).

### 3.2 Trait correlations

Pearson correlations showed that *g*_s_ was negatively correlated with iWUE and Ψ_s, pd_, and iWUE and Ψ_s, pd_ were positively correlated. SLA was negatively correlated with Ψ_s, pd_ and SD, but positively correlated with GL, GW, and SA. H_v_ was positively correlated with Ψ_s, pd_ and RWC_leaf_. Ψ_s, pd_ was positively correlated with SD, but negatively correlated with GL, GW, and SA. RWC_leaf_ was negatively correlated with GW and SA. SD was negatively correlated with GL, GW, and SA, while GL, GW, and SA were positively correlated (**Figure 2**).

**Figure 2:**
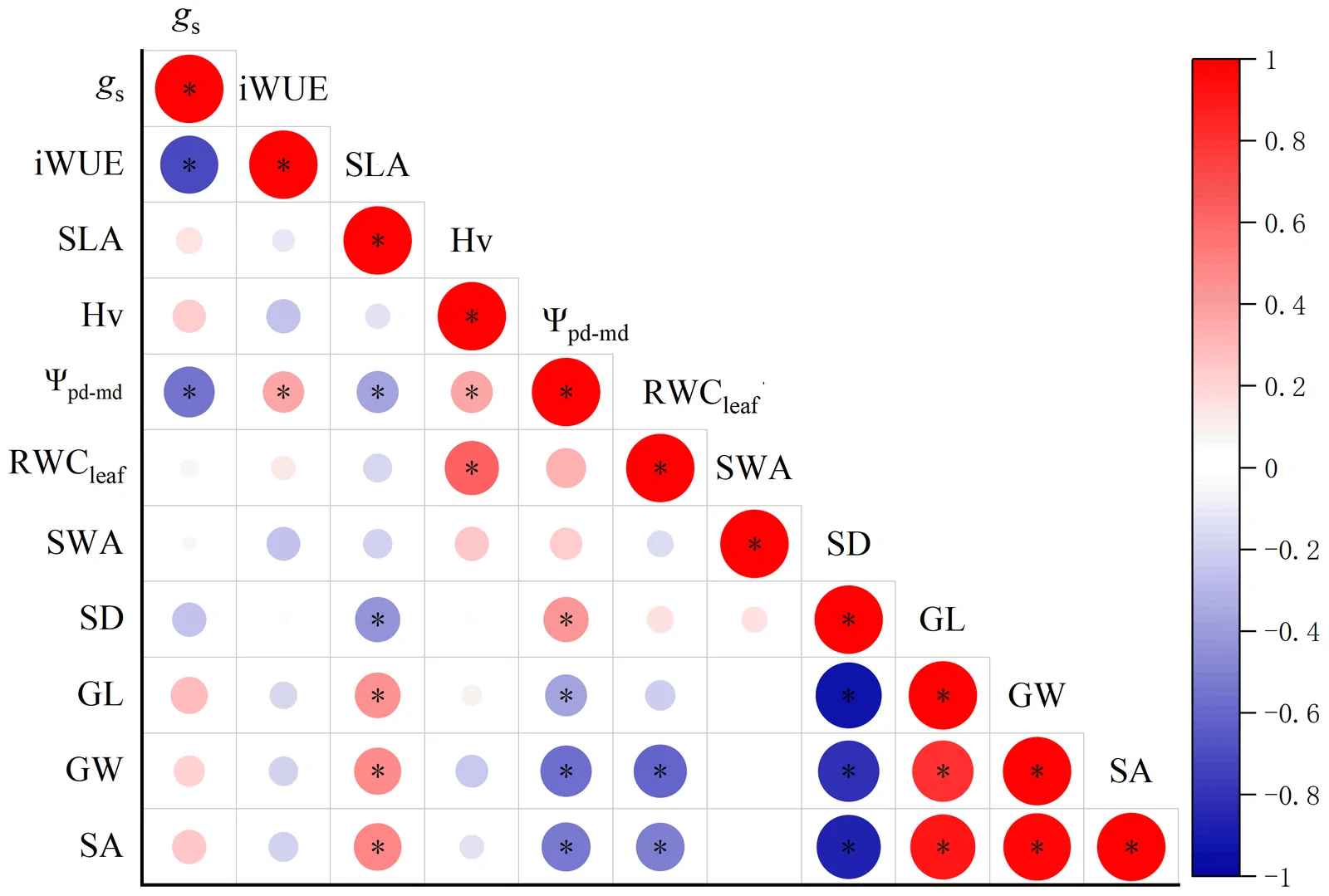
Pearson correlations among measured plant functional traits. Significant correlations are indicated with asterisks (*P* < 0.05). *g*_s_, Stomatal conductance (mmol m^-2^ s^-1^); iWUE, Instantaneous water use efficiency (mmol mol^-1^); SLA, Specific leaf area (cm^2^ g^-1^); H_v_, Huber value; Ψ_pd-md_, Difference in leaf water potential between predawn and midday (MPa); RWC_leaf_, Relative water content of leaf; SWA, Sapwood area (cm^2^); SD, Stomatal density (mm^-2^); GL, Guard cell length (µm); GW, Guard cell width (µm); SA, Stomatal area (µm^2^).

The results of principal components analysis are presented in **Figure 3**. The PCA results showed that PC1 and PC2 cumulatively explained 61.7% of the total variance (43.8% and 17.9%, respectively) (**Figure 3**). The contributions of GW, SA, GL and SD were all over 10%, respectively. In PC1 axis, GW, SA, GL and Ψ_pd-md_ had the main positive contribution while SD had the main positive contribution. In the PC2 axis, iWUE had the main positive contribution while Hv and *g*_s_ had the main negative contribution.

**Figure 3:**
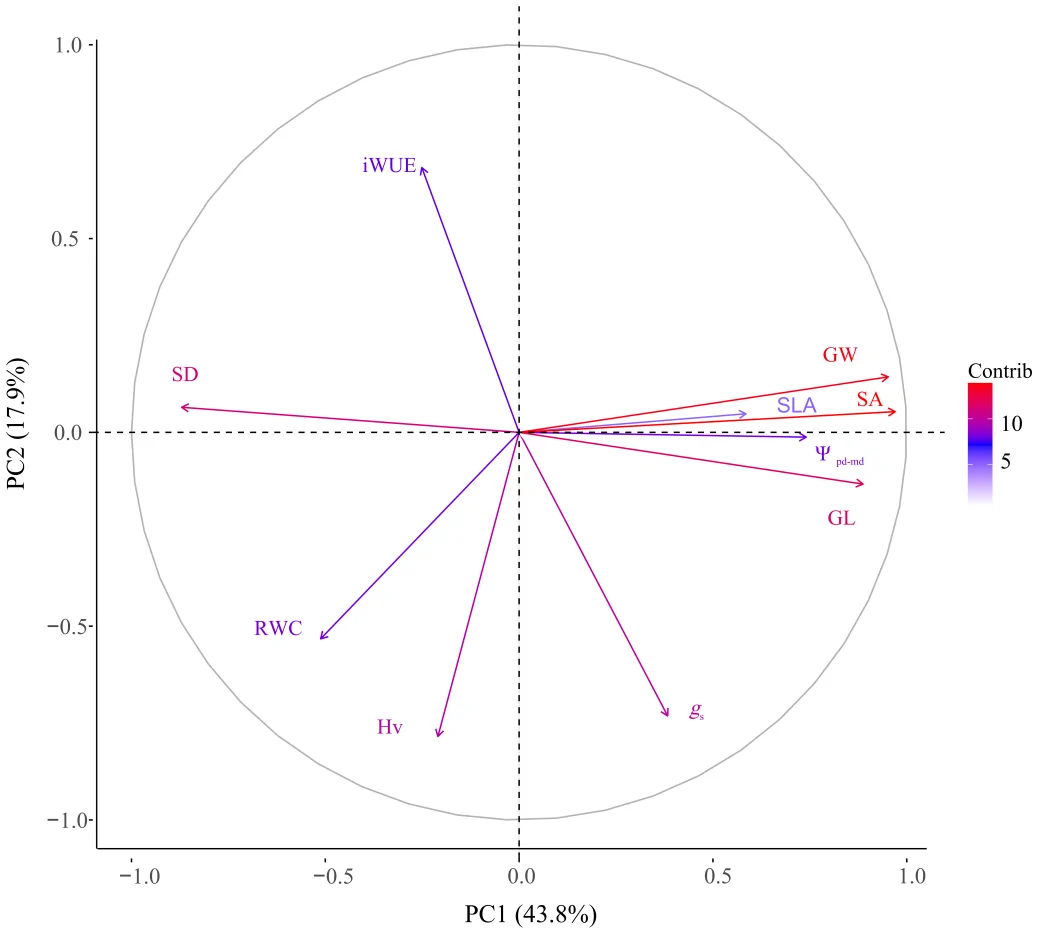
Principal components analysis on 10 functional traits of the four oak species. Trait abbreviations are given in **Figure 2**.

For *Q. dentata*, iWUE, Ψ_s, pd_ and SD were high, while *g*_s_, SLA, H_v_, SWA, GL, GW and SA were low (**Figures 4A**, **1**). For *Q. rubra*, iWUE, GL, GW and SA were high, while *g*_s_, H_v_, RWC_leaf_, SWA and SD were low (**Figures 4B**, **1**). For *Q. robur*, H_v_, Ψ_s, pd_, RWC_leaf_, and GL were high, while SWA and SD were low (**Figures 4C**, **1**). For *Q. palustris*, *g*_s_, SLA, SWA, GL, GW and SA were high, while iWUE, H_v_, Ψ_s, pd_, RWC_leaf_ and SD were low (**Figures 4D**, **1**).

**Figure 4:**
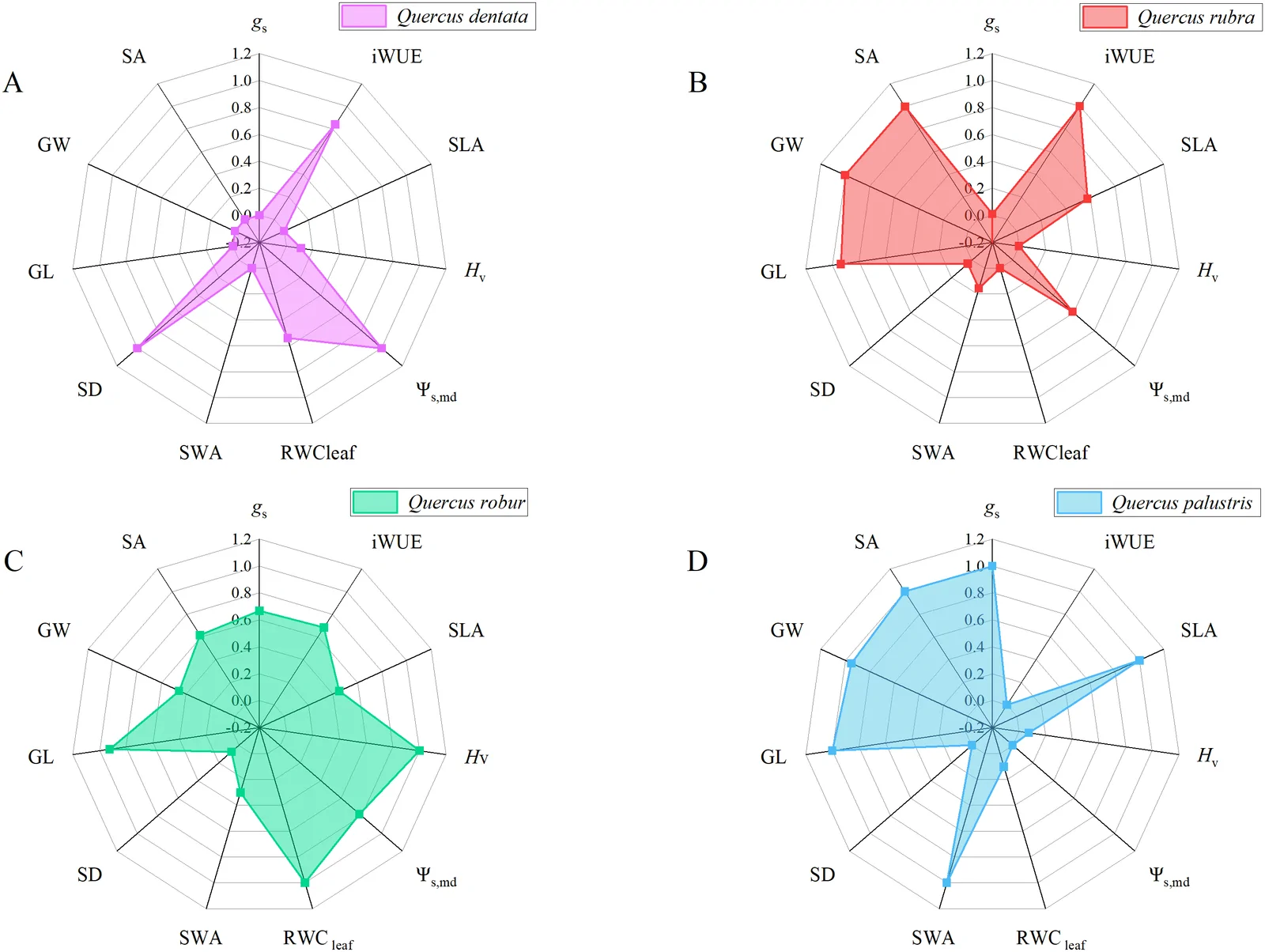
Radar chart for key plant functional traits among four oak species (**A**, Q. dentata; **B**, Q. rubra; **C**, Q. robur; **D**, Q. palustris). Data were standardized for deviation. Trait abbreviations are given in **Figure 2**.

## 4 Discussion

### 4.1 Trait coordination and trade-offs

Plant functional traits were always coordinated/traded off to respond to environmental variations systematically (Brown et al., 2022). According to the Pearson correlations, we found that leaf gas exchange rates were negatively correlated to iWUE (**Figure 2**), indicating a trade-off between gas exchange rate and water use efficiency during carbon assimilation. Besides, faster leaf gas exchange was associated with greater diurnal fluctuations in water potential, posing a potential risk to hydraulic integrity. It’s known as the core trade-off determining leaf-level hydraulic strategy to maintain carbon assimilation vs avoid hydraulic risk (Kannenberg et al., 2019). TLP was known as the capacity to maintain turgor pressure through osmotic adjustment to improve drought tolerance, and showed a higher degree of plasticity that is mainly influenced by environmental factors (Zhu et al., 2018; Xu et al., 2021; Bartlett et al., 2016). However, there was no significant correlation between gas exchange rates and TLP in this study (**Figure 2**). Our findings showed that these four oak species with faster leaf gas exchanges had no need to sacrifice the maintain of cell turgor pressure (quantified by TLP). Leaf turgor determines, in large part, stomatal behavior, but if the guard cells could have efficient exchange of ions and osmolytes, hence requiring a smaller change in turgor when changing pore size (Al‐Salman et al., 2023). Notably, P50 was another key functional trait determining species drought tolerance, used as an indicator of embolism resistance, and previous evidences showed that the faster resource use strategy always coupled higher P50 values (less embolism-resisted) (Kannenberg et al., 2019; Guillemot et al., 2022).

The morphological traits could also provide insight into species’ carbon allocation patterns supporting faster strategy. The Huber value reflected carbon allocation to xylem investment and expected gains obtained by leaf display and, playing a key role in mediating leaf physiology and hydraulics (Xu et al., 2021; Mencuccini et al., 2019). Higher Hv could permit higher leaf water content, and thus sustain more stable leaf water potentials (less negative) (**Figure 2**). Capacity to conduct water to the canopy could be increased by deploying a larger sapwood area at a given total leaf area (higher Hv) (Gleason et al., 2012; Xu et al., 2021), and it was thus a key component of plant hydraulic architecture (Rosas et al., 2019). And higher foliar water use efficiency could also permit lower xylem construction and maintenance costs (Gleason et al., 2016). Thus, large Hv could be seen as a strategy of dehydration avoidance, which helps to reduce the water supply pressure induced by evaporation demand to survive in the face of drought or/and heatwaves.

Specific leaf area (SLA) indicated the leaf-level construction cost of light interception, making it a key trait that influences leaf life span and plant growth, and had been proposed as a correlate or predictor of survival in the face of abiotic and biotic hazards (Bartlett et al., 2012; Díaz et al., 2016; Griebel et al., 2023). Some studies had shown negative correlations between SLA and stomatal density within and among species (Meinzer et al., 2017). In the present study, lower SLA was linked to smaller guard cells with high density, and less negative water potentials (**Figure 2**). Species with ‘conservative’ leaves (low-SLA) should provide fitness benefits in dry/hot environments (Aguirre-Gutiérrez et al., 2025), since higher stomatal density could reduce the water transport pathway from xylem to stomata, promoting more efficient hydraulic supply and maintaining leaf water potential stability (Díaz et al., 2016; Ramírez-Valiente et al., 2010; Al‐Salman et al., 2023). Moreover, leaves with denser stomata responded more quickly and/or strongly, conferring greater tolerance of a variety of stresses (Henry et al., 2019; Coupel-Ledru et al., 2024). However, the conservative species, as water savers, should maintain limited photosynthetic activity and carbon gain. Accordingly, different species could range along a spectrum from lower SLA and higher dehydration avoidance, to larger SLA and faster resource usage, while always satisfying the optimization criterion (Prentice et al., 2014).

### 4.2 Hydraulic strategies among four oak species

*Quercus dentata*, a native oak species, exhibited denser stomata with smaller size, less negative midday water potential and higher iWUE. Accordingly, we defined *Q. dentata* a conservative strategy, along with a promising capacity of drought resilience. Small stomata can open and close more rapidly and their general association with high densities provides the capacity for effective decreases in the stomatal conductance of a leaf, minimizing water loss during unfavorable conditions for dehydration avoidance. Instantaneous water-use efficiency (iWUE) was an important indicator of plant physiological response to environmental changes. In our study, *Q. dentata* conducted high iWUE to reduce water loss during photosynthesis, and thus maintained the water potential less fluctuated, consistent with the theory that the balance between carbon assimilation and the control of water loss was optimized for strategies associated with stress resilience (Davies, 2019; Liu et al., 2016).

Notably, *Q*. *rubra*, an exotic red oak originating from eastern North America, displayed a conservative strategy in our study, showing high iWUE and relatively stable leaf water potential, with less dense stomata in large size. Previous researches showed that *Q*. *rubra* also adopted conservative strategy in the USA, such as maintaining photosynthetic capacity at the drier site while minimizing transpirational water loss (Turnbull et al., 2002), reducing leaf area and hydraulic conductivity to responding to shade and drought (Shumway et al., 1993), confirming a rapid response to relief of stresses (Weber and Gates, 1990). The apparent adaptation was considered as an important factor regulating species performance. Our present results could help advance our understanding of hydraulic performance in exotic species might allow species to grow and survive in arid and semiarid areas that are predicted to become drier and warmer with climate change (Yao et al., 2021).

*Quercus robur* allocated more carbon to sapwood to supply adequate water for leaves to maintain water potential stable, considered to be drought-tolerant. As a major oak species in Central Europe, *Q*. *robur* had been concerned in many studies, but performed differently. Some studies found *Q*. *robur* a highly hygrophilous oak (Kusi and Karsai, 2020), tended to be highly sensitive to drought, where non-stomatal limitations contributed to the photosynthesis decline (Arend et al., 2013; Brüggemann et al., 2002). Yet, some other studies found high resistance and resilience to drought in *Q*. *robur*, with lower specific leaf area and slow growth rate (Morecroft and Roberts, 1999; Svensson et al., 2023). In a warmer and drier future, those species in rapid responses to environmental variations would play an important role in adapting to climate change due to their suitability for various kinds of mixed forest management and potential to store much carbon (Svensson et al., 2023).

*Quercus palustris* was a bottomland oak species originated from North America, known as a wet-tolerant species (Kusi and Karsai, 2020). In the present study, *Q. palustris* had leaves with fast gas exchanges, low iWUE and high SLA, confirming its ability to thrives well in wet habitats. Leaves with high values of SLA were cheaply constructed and representative of fast leaf economics, always associated with progressive growth, less investment in leaf protection and decreased leaf longevity (Dayrell et al., 2018; Díaz et al., 2016). Besides, high cross-sectional area of stem could transport water faster (Cavender-Bares and Holbrook, 2001). Thus species occurring in wetter habitats were able to sustain higher photosynthetic and growth rates under favorable conditions of water, which was considered to be beneficial in terms of carbon, water and nutrient balance since they can maximize carbon uptake and nutrient use when water availability was not limiting (Ramírez-Valiente and Cavender-Bares, 2017). However, we observed fluctuated daily water potential in *Q. palustris* (**Figure 4D**), pointing to a potential of less resistance to dryness. Compared to previous studies in North America, *Q. palustris* thrived well both in bottomland (moist) and upland (dry) habitats, showing great local adaptation to detect environmental changes and produce appropriate plastic responses to cope with those changes (Kusi and Karsai, 2020).

Compared to the native *Q. dentata*, the other three exotic oak species had larger stomata in low density. Stomata are widely recognized as the first line of defense to control transpiration, which played a great role in maintaining leaf temperature within an optimal range and stabilizing the water potential gradient from roots to leaves (Jones, 1998; Holloway-Phillips, 2020). Large stomata allowed for rapid opening and closure, and so might constitute an adaptation to high temperatures and adequate water availability, confirming the competition advantages (Abrams, 1990). While smaller, denser stomata were associated with conservative strategy in water acquisition and usage (Al‐Salman et al., 2023). Smaller stomata had a greater surface area to volume ratio, facilitating ion exchange and thus stronger and faster movements in response to changing irradiance and leaf hydration status (Henry et al., 2019). In conclusion, the trade-off between stomatal density and size reflected the plasticity of stomatal morphology and further long-term adaptation to the local environment.

## 5 Conclusion

We demonstrated the likely potential of well-adaptation to various water conditions for the four studied oak species. Our results showed that the native *Q*. *dentata* was conservative and drought-resistant, while *Q*. *rubra* was conservative and rapid-responsive, *Q*. *robur* was potentially drought-tolerant, and *Q*. *palustris* employed a less drought-resistant but more competitive strategy. Consequently, understanding the factors that limit seedling and sapling establishment, especially when these factors are themselves perturbed by climate change, is central to both our wider understanding of plant community response to climate change and nature-based solutions to mitigate its effects. Integrating these findings into global conservation efforts will lead to the development of adaptive management practices that foster tree species with better adaptation to environmental variations. Taken together, *Q*. *rubra* and *Q*. *robur* could adapt well like *Q*. *dentata*, a native oak species that well-adapted to warm-temperate region. However, modest irrigation may promote growth in *Q*. *palustris* stands on sites in north China, where seasonal dryness is a limiting factor in natural ecosystems. Future studies should quantify a broader set of traits, including xylem embolism resistance, leaf hydraulic conductance, stem-specific hydraulic conductivity, non-structural carbohydrates, nighttime transpiration, and stomatal kinetics, under both controlled drought and field conditions. It will also be important to test whether the conservative-acquisitive axis identified here is stable across ontogeny, soil types, and more severe drought, and to link these traits to survival, growth, and establishment in mixed-species plantations.

## Data Availability

The raw data supporting the conclusions of this article will be made available by the authors, without undue reservation.
