# Supplementary material for: Differential hydraulic and carbon economic strategies among four oak species
Source: Front Plant Sci. 2026 Oct 15;17:1983948. doi: 10.3389/fpls.2026.1983948 (PMC13716799; doi:10.3389/fpls.2026.1983948)
Supplement: Supplementary file 1 [file SupplementaryFile1.docx]

Table S1.Mean height and diameter at breast height (DBH) of the four oak species

| Species | Origin/status | Acorn source | n | Height(m) | DBH(cm) |
| --- | --- | --- | --- | --- | --- |
| *Q. dentata* | Native to China | Dongtai, Jiangsu | 8 | 3.53 ± 0.26 | 5.95 ± 0.20 |
| *Q. rubra* | Exotic | Dongtai, Jiangsu | 8 | 3.78 ± 0.14 | 6.46 ± 0.17 |
| *Q. robur* | Exotic | Dongtai, Jiangsu | 8 | 4.19 ± 0.34 | 5.11 ± 0.24 |
| *Q. palustris* | Exotic | Dongtai, Jiangsu | 8 | 4.44 ± 0.09 | 5.25 ± 0.08 |

Table S2 Mean values of plant functional traits among four oak species

|  |  | *Q. dentata* | *Q. rubra* | *Q. robur* | *Q. palustris* |
| --- | --- | --- | --- | --- | --- |
| Leaf gas exchange traits | *E* | 2.95 ± 0.39 a | 2.61 ± 0.34 a | 4.55 ± 0.55 b | 5.23 ± 0.71 b |
|  | *A* | 14.53 ± 1.16 a | 14.57 ± 1.10 a | 22.54 ± 1.34 b | 19.46 ± 1.33 b |
|  | *C*_i_ | 221.31 ± 26.10 a | 231.69 ± 13.38 a | 230.47 ± 18.68 a | 272.12 ± 15.75 a |
|  | *g*_s_ | 0.19 ± 0.02 ab | 0.17 ± 0.03 a | 0.27 ± 0.05 ab | 0.33 ± 0.06 b |
|  | iWUE | 5.63 ± 0.89 b | 5.94 ± 0.42 b | 5.32 ± 0.45 b | 3.99 ± 0.36 a |
| Hydraulic traits | Ψ_s,md_ | -0.08 ± 0.01 a | -0.11 ± 0.01 a | -0.09 ± 0.01 a | -0.14 ± 0.01 b |
|  | Ψ_s,pd_ | -0.028 ± 0.003 a | -0.022 ± 0.001 a | -0.023 ± 0.002 a | -0.027 ± 0.004 a |
|  | Ψ_TLP_ | -1.11 ± 0.19 a | -1.44 ± 0.16 a | -1.48 ± 0.15 a | -1.27 ± 0.12 a |
|  | RWC_leaf_ | 1.33 ± 0.02 b | 1.09 ± 0.03 a | 1.53 ± 0.02 c | 1.14 ± 0.01 a |
|  | SWA | 0.40 ± 0.05 | 0.41 ± 0.04 | 0.42 ± 0.03 | 0.46 ± 0.05 |
|  | H_v_ | 3.75 ± 0.49 a | 3.20 ± 0.45 a | 7.65 ± 0.99 b | 3.59 ± 0.64 a |
| Structural traits | SLA | 91.88 ± 2.65 a | 103.08 ± 6.44 ab | 100.65 ± 2.21 ab | 111.34 ± 1.96 b |
|  | SD | 0.40 ± 0.02 b | 0.18 ± 0.01 a | 0.19 ± 0.01 a | 0.17 ±0.01 a |
|  | SA | 4038.48 ± 201.54a | 10371.33 ± 316.72 c | 7952.34 ± 322.42 b | 10392.01 ± 396.94 c |
|  | GL | 129.88 ± 4.98 a | 192.40 ± 1.64 b | 191.55 ± 3.99 b | 197.02 ± 3.63 b |
|  | GW | 39.51 ± 0.74 a | 68.60 ± 1.64 c | 52.80 ± 1.41 b | 67.13 ± 1.88 c |

The data in the table are the mean ± SE (*n* = 8). Significant differences (*P*<0.05) between different species by Duncan’s test are indicated with different letters. *E,* Transpiration rate (mmol m^-2^ s^-1^); *A,* Net photosynthetic rate (μmol m^-2^ s^-1^); *C*_i,_ Intercellular CO_2_ concentration (μmol mol^-1^); *g*_s,_ Stomatal conductance (mol m^-2^ s^-1^); iWUE, Instantaneous water use efficiency (mmol mol^-1^); Ψ_s,md,_ Leaf midday water potential (MPa); Ψ_s,pd_; Leaf predawn water potential (MPa); Ψ_TLP_, Osmotic potential at the turgor loss point (MPa); RWC_leaf_, Relative water content of leaf; SWA, Sapwood area (mm^2^); H_v_, Huber value; SLA, Specific leaf area (cm^2^ g^-1^); SD, Stomatal density; SA, Stomatal area (mm^-2^); GL, Guard cell length (mm); GW, Guard cell width (mm)
